# Impact of the implementation of the AAN epilepsy quality measures on the medical records in a university hospital

**DOI:** 10.1186/1471-2377-13-112

**Published:** 2013-08-28

**Authors:** J Miguel Cisneros-Franco, Marco A Díaz-Torres, Juan B Rodríguez-Castañeda, Alejandro Martínez-Silva, Mildred A Gutierrez-Herrera, Daniel San-Juan

**Affiliations:** 1 Neurology Service, Universidad Autónoma de Nuevo León, Av. Madero esq. Dr. J.E. González s/n, Monterrey, Nuevo Leon 64460, Mexico; 2 Present address: McGill University Integrated Program in Neuroscience, 3801 University St, Rm 744, Montréal, QC H3A 2B4, Canada; 3 School of Medicine, Universidad Autónoma de Nuevo León, Av. Madero esq. Dr. Aguirre Pequeño s/n, Monterrey, Nuevo Leon 64460, Mexico; 4 Neurophysiology Department, Instituto Nacional de Neurología y Neurocirugía, Insurgentes Sur 3877, Tlalpan, Mexico City 14269, Mexico

**Keywords:** Academic medical center, Quality of health care, Adult epilepsy, Health education, AAN epilepsy quality measures, General practitioners

## Abstract

**Background:**

The American Academy of Neurology (AAN) suggested eight quality measures to be observed at every patient visit. The aim of this work is to compare the percentage of documentation of each measure before and after the implementation of a new worksheet in a third-level center.

**Methods:**

Quasi-experimental study including medical records filled by medical school seniors and junior residents supervised by an epileptologist. The authors surveyed 80 consecutive charts of people with epilepsy who were seen in the outpatient clinic before and after the intervention. McNemar change test was used to compare the percentages of documentation of each quality measure–i.e., seizure type and frequency, etiology, EEG, MRI/CT head scans, AED side effects, surgical therapy referral, safety counseling, preconception counseling–and physical exam. Each quality measure was considered to be fulfilled only if it was assessed and properly recorded.

**Results:**

Mean age was 35(±13) years, 55% women, mean epilepsy onset at age 18(±15), 82% presented with partial-onset seizures. The reporting rate improved for all quality measures (previous *vs* new), reaching statistical significance for: seizure type 80*vs*94% (p < 0.05), AED side effects 8*vs*24%, etiology 66*vs*88% (p < 0.01), safety counseling 5*vs*64%, preconception counseling 4*vs*20%, and physical exam 63*vs*94% (p < 0.001).

**Conclusion:**

A quality-oriented epilepsy worksheet led to a better practice standardization and documentation of AAN standards for diagnostic and counseling purposes. Further evaluations should be undertaken to assess the impact on medical education and patient care.

## Background

The burden of epilepsy remains a public health issue worldwide, particularly in developing countries, where 90% of people with epilepsy (PWE) live. These subjects are often misdiagnosed or left untreated, due to economical or educational deficiencies [1]. Certainly, physicians can benefit individual patients by following guidelines and practice parameters [2]. However, quality of care, defined as the implementation of policies in populations to improve care [3], has been difficult to assess, due to the lack of widespread quality measures specifically targeted to PWE.

For this reason, the American Academy of Neurology (AAN), through the Physician Consortium for Performance Improvement, suggested eight quality measures to be observed [4], in an effort to standardize the care for PWE. The first four evidence-based measures intend to guide the clinician towards a proper diagnostic approach and, subsequently, an adequate treatment: (1) determination of seizure type/frequency, (2) etiology, and review/order of (3) EEG and (4) neuroimaging studies. The last four measures focus on (5) surgical referral, (6) counseling about drug side effects, (7) safety issues and (8) reproductive health.

Adherence to these quality measures has been reported both by a recent survey among neurologists [5], and by a retrospective study focused on pediatric neurologists [6]; but information regarding its use by non-specialist physicians or medical students is lacking. We deemed the appearance of these measures as an opportunity to improve patient care in our University Hospital, where medical students are directly involved in the function of the Epilepsy Clinic. With this purpose, we developed a new quality-oriented epilepsy worksheet, as a user-friendly guide for medical trainees, taking into account the AAN quality measures.

The first aim of this study is to compare the percentage change of documentation of adherence to each AAN quality measure, before and after the implementation of a new worksheet in a third-level University Hospital in north-eastern Mexico. We also analyzed the documentation of physical exam findings as an additional measure, given its clinical relevance [7] and the educational nature of our institution. Hence, the second aim of the study is to compare the percentage change of documentation of physical exam that followed the implementation of the worksheet. We expected to observe an improvement in the documentation of the AAN quality measures and physical exam with the use of this quality-oriented worksheet.

## Methods

### Location

Outpatient neurology clinic, Hospital Universitario ‘Dr. José E. González’, Monterrey, Mexico. This hospital is the main referral centre of the public health system in north-eastern Mexico, a region comprising 3 states with a combined population of 11,042,149 inhabitants as of 2012.

### Type of study

Prospective, quasi-experimental.

### Standard protocol approvals, registrations, and patients consents

The study was approved by our Institutional Review Board, *Comité de Ética y Comité de Investigación de la Facultad de Medicina y Hospital Universitario ‘Dr. José Eleuterio González’*.

### Paticipants and clinical information

Data from 175 consecutive PWE, filled by medical school seniors and junior residents. A total of 60 medical students and two neurology residents were involved. These trainees see patients initially in groups of two or three each, and are responsible for writing a note, followed by a revisit with an attending epileptologist. The clinical variables we recorded were: age, gender, age at epilepsy onset, duration of epilepsy, seizure frequency, number of antiepileptic drugs (AEDs) currently used, number of AEDs used during their lifetimes (whether in monotherapy or polytherapy).

### Instruments

In the University Hospital, outpatient notes follow the traditional SOAP (subjective, objective, assessment, and plan) method, without format differences between services. An epileptologist and a recent medical graduate developed a new quality-oriented epilepsy worksheet (see Additional file 1), tailored to the needs of our outpatient epilepsy clinic; where the subjects are scheduled for follow-up visits at least each 4 months. We decided to include all the essential items that should comprise a complete anamnesis of a person with epilepsy, instead of focusing only on the 8 AAN recommendations. Over a period of ~1 month, a preliminary version of the worksheet was used by two attending physicians (a neurologist and an epileptologist), who took note of the missing items that should be further included. Once the design of the worksheet was finalized, its use was explained to the trainees on their first day of a 2-week rotation, and a sample filled document was placed on every desk for reference. No other educational session was undertaken.

At the end of the six-month period following the introduction of the new worksheet, the medical charts were revised, considering a quality measure to be fulfilled only if it was assessed and properly recorded. For any given chart, the same person was responsible for reviewing the patient notes corresponding to the visit before and after the intervention–i.e., the introduction of the new worksheet–. Seizure frequency in a patient with multiple seizure types had to be recorded separately for each one (measure #1). Etiology needed to be classified as recommended by the International League Against Epilepsy (measure #2) [8]. MRI–rather than CT scan–and EEG are warranted and readily available at our institution, to assess the concordance between seizure semiology, electrographic and neuroimaging studies. If EEG and/or MRI studies (measures #3 and #4, respectively) have already been requested, the result had to be noted down. The annotations regarding physical exam, AEDs side effects (measure #5), and surgical therapy referral (measure #6), as well as the discussion of safety issues (measure #7) and reproductive health (measure #8) required a description of these. Counseling about epilepsy safety issues included: clarification of doubts regarding epilepsy, explanation of what to do during a seizure, review of seizure diary and/or providing a new diary, and invitation to the monthly meeting of the epilepsy support group. Counseling for women of childbearing potential included resolving doubts about pregnancy and interrogation about contraception.

### Statistical analysis

We performed descriptive and non-parametric analyses with SPSS software, version 17.0. McNemar change test was used to compare the percentages of documentation for each AAN quality measure and physical exam, before and after the intervention.

## Results

### Descriptive statistics

One hundred and seventy-five consecutive medical charts of PWE were evaluated: 103 charts had at least one SOAP note, 152 had at least one new worksheet; and 80 charts had both types of notes available, thus being amenable for direct comparison and analysis. There were not statistically significant differences, in the demographics or in the percentages of compliance with the quality measures, between the whole sample and the third subset used for comparison (n = 80), to which we refer now in this manuscript. This subset included patients aged 17–73 years (34.7 ± 13 years, mean ± SD), 44 females and 36 males (45%). Thirty-four women were considered of childbearing potential (12–44 years old) as per AAN standards [4]. Partial-onset seizures were the most common type of epilepsy, while the main etiologic classification corresponded to structural/metabolic epilepsies. Table 1 shows the clinical data.

**Table 1:** Summary of clinical data from patients

	**Comparison group (n = 80)**				
	**F:M 55:45%**				
	**Range**	**Mean**	**SD**		
Age	17-73	34.7	13	Seizure onset	
Age at epilepsy onset	1-68	18.2	14.7	Partial	82.4%
				Generalized	17.6%
Duration of epilepsy (years)	1-32	14.9	12.6		
Seizure frequency (month)	0-30	2.2	4.2	Etiology	
				Genetic	18.7%
Current AEDs	1-4	1.2	2.1	Structural/metabolic	46.3%
Lifetime AEDs	1-8	4.7	2.5	Unknown	35%

AED: antiepileptic drugs; F: female, M: male; SD: standard deviation.

Regarding the assessment and appropriate recording of clinical data for each visit, we found an improvement in the documentation of all quality measures after the implementation of the new worksheet. Percentage changes are shown in Table 2.

**Table 2:** Documentation of the AAN epilepsy quality measures and physical exam before and after the introduction of a quality-oriented worksheet in a University Hospital

**Quality measure**	**Documentation % (n)**				**χ**^**2**^	**p value**	
	**Before**		**After**				
Seizure type	81.25	(65)	93.75	(75)	4.5	0.0339	*
Seizure frequency	50	(40)	63.75	(51)	2.70	0.1002	
Etiology	66.25	(53)	87.5	(70)	9.48	0.0021	**
EEG	63.75	(51)	76.25	(61)	2.89	0.089	
Neuroimaging	42.5	(34)	56.25	(45)	3.23	0.0725	
AED side effects	7.5	(6)	27.5	(22)	9.38	0.0022	**
Surgical referral	2.56	(1)	12.82	(5)	2.25	0.1336	
Safety counseling	3.75	(3)	62.5	(50)	43.18	<0.0001	^@^
PCC	11.76	(4)	47.05	(16)	10.08	<0.0001	^@^
Physical exam	63.75	(51)	93.75	(75)	21.33	<0.0001	^@^

PCC: preconception counseling. *p < 0.05; **p < 0.01; @p < 0.0001.

The percentages quoted for surgical therapy referral were determined according to the most recent definition of drug resistant epilepsy [9], identified in 39 of the 80 patients considered for comparison. Before the intervention, one patient with a brain tumor presenting with epilepsy was referred to neurosurgery. After the intervention, this group included five patients: two with mesial temporal lobe epilepsy and hippocampal sclerosis, one with cortical dysplasia, and two with brain tumors. Among the patients for which information regarding AED side effects was reported (28%, n = 22), four had an abnormal physical exam that evidenced side effects that have not been reported previously.

### Non-parametric statistics

McNemar change test (χ^2^ critical value = 3.84; 1 degree of freedom; 95% confidence interval) showed statistical significance for seizure type (p < 0.05), etiology (p < 0.01), AED side effects (p < 0.01), safety counseling (p < 0.001), preconception counseling (p < 0.001), and physical exam (p < 0.001) (Table 2).

## Discussion

The AAN quality measures serve as a road-map that can be used for both primary care physicians and neurologists [4], although its use has been investigated only among specialists [5,6]. A ≥80% adherence for seizure type/frequency (measure #1), EEG (measure #3), and neuroimaging (measure #4) was reported independently in both a survey of neurologists [5] and a single tertiary care pediatric epilepsy center [6]. To our knowledge, this is the first study to assess the adherence to the AAN quality measures in an academic setting. As we anticipated, there was an improvement in the documentation of all measures after the implementation of the new quality-oriented worksheet.

We decided to analyze seizure type and frequency separately, because we noticed that asking one does not necessarily warrants asking the other. For seizure type, the baseline figure was similar to that reported in another teaching hospital [10], and improved after the intervention in our study. This difference could be attributable to the fact that we provided trainees with a seizure type checklist (see Additional file 1). In doing so, we aimed to encourage them to classify each seizure using the available information.

Both seizure type and frequency of seizures contribute to define the epileptic syndrome; while seizure frequency also aids to assess its severity. Moreover, in our environment–open population with a low rate of social insurance–, seizure frequency may serve as an indirect measure of compliance [11]. Although documentation of seizure frequency improved, we agree with the observations by Ulloa & Gilliam [12], that patient self-reported seizure count does not necessarily provide accurate information [13].

The clinician must recognize etiology–even for follow-up visits–, as a dynamic entity that may change in the light of new tests or re-interrogation of the patient [14]. For this reason, we placed the field for etiology after that assigned to the commentaries of the attending physician, inviting the trainees to reflect on the data they had already gathered.

Physicians tend to direct their efforts towards seizure control, rather than active supervision of AED side effects [12]. In this study, side effects were documented in one of every four PWE, although they have been reported in up to 50% of AED users [15].

The charts of five patients–with and without structural lesions–included notes of referral for pre-surgical evaluation, as opposed to one patient before the intervention–who had a structural lesion. It is important to note that, despite Class I evidence, people with non-lesional drug resistant epilepsy still experience an unacceptable delay (more than 20 years) in referral for surgical management [16].

We found a ten-fold increase in registered safety counseling, a broad, unclear and controversial topic [12]. Efforts were directed mainly towards education regarding appropriate behaviors during a seizure and accident prevention, leaving other themes for the monthly meeting of the Epilepsy support group.

Preconception counseling showed significant improvement, yet it remains an important challenge. The clinician needs to go beyond the prevention of pregnancy: it is necessary to discuss with the woman of childbearing potential how to plan her pregnancy, doing the pertinent AEDs and supplements adjustments; even if current evidence concerning the actual effectiveness of counseling is inconclusive [17].

Physical exam is considered in the guidelines of the National Association of Epilepsy Centers [18], but only during seizures. However, interictal examination is opportune, whether the patient presents with new-onset seizures–to look for any abnormality suggesting the etiology or associated conditions–or during follow-up visits–to assess associated injuries, cognitive deficits, and AEDs side effects [7]. In our study, four patients (18%) of those with recorded information about AED side effects had an abnormal physical exam. Moreover, in the setting of a University Hospital, the practice of neurological examination presents as a unique opportunity for the medical trainees to develop the skills needed to perform their duties as primary-care physicians. This is of special importance, given that much of the care of PWE is delivered by primary care physicians [3]. In this regard, any centre that brings together health care providers with different levels of training has the invaluable opportunity to encourage them to address the social, behavioral and psychological issues faced by PWE. These issues remain an unresolved matter in epilepsy and may influence seizure outcome [19], whether a patient is managed by a primary-care physician or a neurologist [20].

This study has some limitations. First, the figures quoted in this study do not necessarily reflect whether or not the matters were discussed with the patient. Instead, they reflect whether or not there was documentary evidence of such discussion. Therefore, the fact that the study looked at the documentation of the quality measures may underestimate its impact on the quality of healthcare given to PWE. Anyhow, it is evident that hospitals and external quality control agencies rely on medical records, as opposed to reported verbal discussion, to assess and monitor the quality of care by healthcare professionals [2]. Second, the focus of this paper was not on long-term outcomes of patients. Third, the study is limited by its design: it lacks a control group for comparison between the medical records of patients who have been exposed to the intervention and those who were not.

The improvement in documentation reported in this manuscript may have implications beyond an academic medical centre. Any group or individual physician, regardless of their level of training or workplace, may use this worksheet as a user-friendly template to develop their own materials to help themselves to comply with the epilepsy quality measures. Additionally, the documentation of the AAN epilepsy quality measures can be used as an indicator of the efficacy and quality of care by international certifying agencies focused on patient safety [21]. Our findings increase the external validity of these measures in non-neurologists; however, additional studies are needed to evaluate long-term outcomes of patients. This would be an interesting matter of analysis, in terms of quality of life, epilepsy knowledge, or medication adherence.

## Conclusions

While guidelines, measures and consensus give more or less formal instructions to be followed, it is critical that medical students and residents understand its fundaments. We believe that, only to the extent that these trainees are coached about what to look for and the benefits of doing it, will they have a satisfying educational experience with long-lasting teachings. This work provides an example of how an easy intervention, in a restricted-budget public institution from a developing country, could result effective as a first step to improve the quality of medical care; as it has been shown in other fields of medicine, where standardized worksheets are gaining momentum as safety tools with proven impact on clinical care [22]. Additional studies shall provide insights on the efficacy of similar interventions in the improvement of medical education and, more importantly, the care of people with epilepsy.

## Competing interests

The authors declare that they have no competing interests.

## Authors’ contributions

JMC participated in the design of the study, performed the statistical analysis, and drafted the manuscript. MAD participated in the design and coordination of the study, and helped to draft the manuscript. JBR participated in data acquisition and reviewed the manuscript. AM participated in data acquisition and reviewed the manuscript. MAG participated in the design of the study and reviewed the manuscript. DS drafted and reviewed the manuscript. All authors read and approved the final manuscript.

## Pre-publication history

The pre-publication history for this paper can be accessed here:

http://www.biomedcentral.com/1471-2377/13/112/prepub

## Supplementary Material

Additional file 1 **Epilepsy clinic worksheet.** Quality-oriented epilepsy worksheet, developed by an epileptologist and a recent medical graduate, designed to be used by medical students and residents in the UANL University Hospital. Click here for file
