# Supplementary material for: Impact of the implementation of the AAN epilepsy quality measures on the medical records in a university hospital
Source: BMC Neurol. 2013 Aug 28;13:112. doi: 10.1186/1471-2377-13-112 (PMC3765766; doi:10.1186/1471-2377-13-112)
Supplement: Additional file 1 — Epilepsy clinic worksheet. Quality-oriented epilepsy worksheet, developed by an epileptologist and a recent medical graduate, designed to be used by medical students and residents in the UANL University Hospital. [file 1471-2377-13-112-S1.pdf]

NEUROLOGIA
HOSPITAL UNIVERSITARIO

Name____
File #____
Age__________ Gender____________ Date______________
Phone____
□ 1st visit □ Follow-up (*start from HPI)

EPILEPSY CLINIC

This format is in compliance with the regulations of the Minister of Health NOM-168-SSA1-1998

Hand dominance: □R □L □A

PMH & familial history relevant for epilepsy (specify)**:**
□FH: ______ □TBI: ______ □Febrile sz: ______ □Status epil: ______ □Neuroinfectious: ______ □ Drug abuse:_______
Pregnancy/Perinatal:____ _______________________________
Psychomotor development & education:____
Other medical/surgical conditions: ____

HPI (including age of onset for each seizure type)

(1st visit: list ALL antiepilpetic drugs ever used)

Seizure type(s)

□SPS Simple partial □ CPS Complex partial □SGS Secondarily Generalized
□GTCS Generalized tonic-clonic seizures
□Absence (Typicla/Atypical) □Tonic □Atonic □Myoclonic □Unclassified

Date of last seizure: ____/____/____ **Predominant time of day**: _______________
Triggered by: □Fever □Sleep deprivation □Menses □Stress □Alcohol □Other(s): ________________________________

AED	Dose (mg)	Freq	Serum lvl		Seizure diary								
					(month)	(seizure type)	(frequency)	(month)	(seizure type)	(frequency)	(month)	(seizure type)	(frequency)

AED side effects: □No □Yes: ____

PE (include superior mental functions/cognitive/mood)

BP_________ HR _________ RR ____________ Weight__________________

Paraclinical studies & referrals (1st visit: list all available studies; follow-up: list new results since last visit)

EEG ____/____/____ ____

EEG ____/____/____ ____

CT ____/____/____ ____

MRI ____/____/____ ____

Others: ____/____/____ ____

Referrals: ____/____/____ ____

EDUCATION ABOUT EPILEPSY (specify the actions performed <u>during this visit</u>)

□Information about the disease, clarification of doubts: ______________________________

□Accident prevention, what to do during a seizure

□Seizure diary □reviewed / □ new provided

□Invitation to the *Epilepsy support group* (last wed of the month, 10 AM)

□Preconception counseling / contraceptive used (12-44 y-o F): ______________________________

Comments by the professor

□Drug-resistant epilepsy: does the patient meet criteria for a clinical trial or surgery?

□The following studies / interconsultations are required:

Etiology: □Genetic □Structural/metabolic □Unknown

Dx impression: (Classification of the syndrome/constellation)**:**

Plan:

Med student____________________ Resident____________________ Professor____________________
